# The Involvement of the RhoA/ROCK Signaling Pathway in Hypersensitivity Reactions Induced by Paclitaxel Injection

**DOI:** 10.3390/ijms20204988

**Published:** 2019-10-09

**Authors:** Chen Pan, Yu-Shi Zhang, Jia-Yin Han, Chun-Ying Li, Yan Yi, Yong Zhao, Lian-Mei Wang, Jing-Zhuo Tian, Su-Yan Liu, Gui-Qin Li, Xiao-Long Li, Zhong Xian, Ai-Hua Liang

**Affiliations:** Key Laboratory of Beijing for Identification and Safety Evaluation of Chinese Medicine, Institute of Chinese Materia Medica, China Academy of Chinese Medical Sciences, Beijing 100700, China; cpan@icmm.ac.cn (C.P.); yszhang@icmm.ac.cn (Y.-S.Z.); jyhan@icmm.ac.cn (J.-Y.H.); cyli@icmm.ac.cn (C.-Y.L.); yyi@icmm.ac.cn (Y.Y.); yzhao@icmm.ac.cn (Y.Z.); lmwang@icmm.ac.cn (L.-M.W.); jztian@icmm.ac.cn (J.-Z.T.); syliu@icmm.ac.cn (S.-Y.L.); liguiqinzys@163.com (G.-Q.L.); hobbyarman@163.com (X.-L.L.); xianzhonglove@126.com (Z.X.)

**Keywords:** paclitaxel injection, hypersensitivity reactions, vascular permeability, histamine release, complement activation, RhoA/ROCK signaling pathway

## Abstract

A high incidence of hypersensitivity reactions (HSRs) largely limits the use of paclitaxel injection. Currently, these reactions are considered to be mediated by histamine release and complement activation. However, the evidence is insufficient and the molecular mechanism involved in paclitaxel injection-induced HSRs is still incompletely understood. In this study, a mice model mimicking vascular hyperpermeability was applied. The vascular leakage induced merely by excipients (polyoxyl 35 castor oil) was equivalent to the reactions evoked by paclitaxel injection under the same conditions. Treatment with paclitaxel injection could cause rapid histamine release. The vascular exudation was dramatically inhibited by pretreatment with a histamine antagonist. No significant change in paclitaxel injection-induced HSRs was observed in complement-deficient and complement-depleted mice. The RhoA/ROCK signaling pathway was activated by paclitaxel injection. Moreover, the ROCK inhibitor showed a protective effect on vascular leakage in the ears and on inflammation in the lungs. In conclusion, this study provided a suitable mice model for investigating the HSRs characterized by vascular hyperpermeability and confirmed the main sensitization of excipients in paclitaxel injection. Histamine release and RhoA/ROCK pathway activation, rather than complement activation, played an important role in paclitaxel injection-induced HSRs. Furthermore, the ROCK inhibitor may provide a potential preventive approach for paclitaxel injection side effects.

## 1. Introduction

Paclitaxel injection, a microtubule-stabilizing agent causing cancer cell death or inducing apoptosis [1], is widely used for the treatment of several malignancies, including ovarian, breast, and non-small cell lung cancers [2]. However, the use of paclitaxel is sometimes limited due to the high incidence of severe hypersensitivity reactions (HSRs) [3] characterized by a rapid onset of generalized urticaria, angioedema, pulmonary edema, dyspnea, hypotension, and tachycardia [4], which are similar to type I hypersensitivity. Approximately 16% to 40% of patients receiving paclitaxel infusions suffer from HSRs, even on the first exposure [5]. Some studies also claim that taxane-induced acute HSRs are not mediated by specific immunoglobulin E (IgE) [6] and have a non-allergic nature [7]. Mast cell degranulation has been consistently confirmed to be one of the causes of paclitaxel injection-induced HSRs [8,9].

Recent studies have shown that polyoxyl 35 castor oil (Cremophor EL), one of the excipients of paclitaxel injection, is partly responsible for paclitaxel injection-induced HSRs by evoking the complement system [10] and histamine release [11] in in vitro studies. The glucocorticoid dexamethasone, at therapeutic concentrations, decreases complement component 3 (C3) and has anti-inflammatory properties [12]. Despite premedication with a combination of corticosteroid and anti-histamine agents, HSRs still occur during paclitaxel infusion in the treatment of solid tumors in 4% of patients, and severe HSRs (≥grade 3) happen in 1% of patients [13]. Hence, we hypothesized that other mechanisms resulting in paclitaxel injection-induced HSRs besides complement activation and histamine release may be present. Due to the lack of in vivo data, novel models mimicking paclitaxel injection HSRs that closely resemble the clinical symptoms are urgently needed.

The symptoms of paclitaxel injection-induced HSRs, including angioedema, generalized urticaria, and pulmonary edema, are related to vascular hyperpermeability. Previous studies suggested that RhoA/ROCK signaling pathway activation is involved in vascular endothelial hyperpermeability in response to various inflammatory factors, such as histamine [14], cytokines [15], or vascular endothelial growth factor [16] by upregulation of active state small GTPase RhoA [17]. Thus, we hypothesized that the RhoA/ROCK signaling pathway might be associated with paclitaxel injection-induced HSRs. 

In this study, we developed a mice model to investigate the HSRs of paclitaxel injection, and evaluated the roles of histamine release, complement activation, and RhoA/ROCK signaling pathway activation on paclitaxel injection-induced HSRs

## 2. Results

### 2.1. First Exposure of Paclitaxel Injection Could Cause Hypersensitivity Reactions, Which Are Mainly Induced by Cremophor EL 

According to the manufacturer’s instructions, the clinical doses of paclitaxel injection recommended for breast cancer treatment are an infusion of 135–175 mg/m^2^ at final concentrations of 0.3 to 1.2 mg/mL. The doses of paclitaxel injection used in the present study in mice (4, 8, and 16 mg/kg) were about 1/8, 1/4, and 1/2 of clinical dosages, according to the conversion between animal doses and human equivalent doses [18]. In the preliminary experiments in mice, we found that paclitaxel injection at doses higher than 20 mg/kg could cause death in most mice.

**Figure 1:**
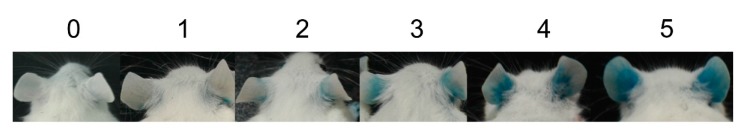
The scoring standard (from “0” to “5”) for evaluating the areas of blue color in ear exudation.

Paclitaxel was slowly intravenous injected (iv) into Institute of Cancer Research (ICR) mice and immediately followed by one 0.8% Evans blue (EB) injection (iv). EB, which binds plasma albumin to form a complex of albumin–EB, has been widely used as a marker for evaluating vascular leakage, and makes vascular permeability visible and measurable [19]. We scored “0” to “5” according to the size of blue area in the ear to evaluate the degree of vascular leakage (Figure 1). 

**Figure 2:**
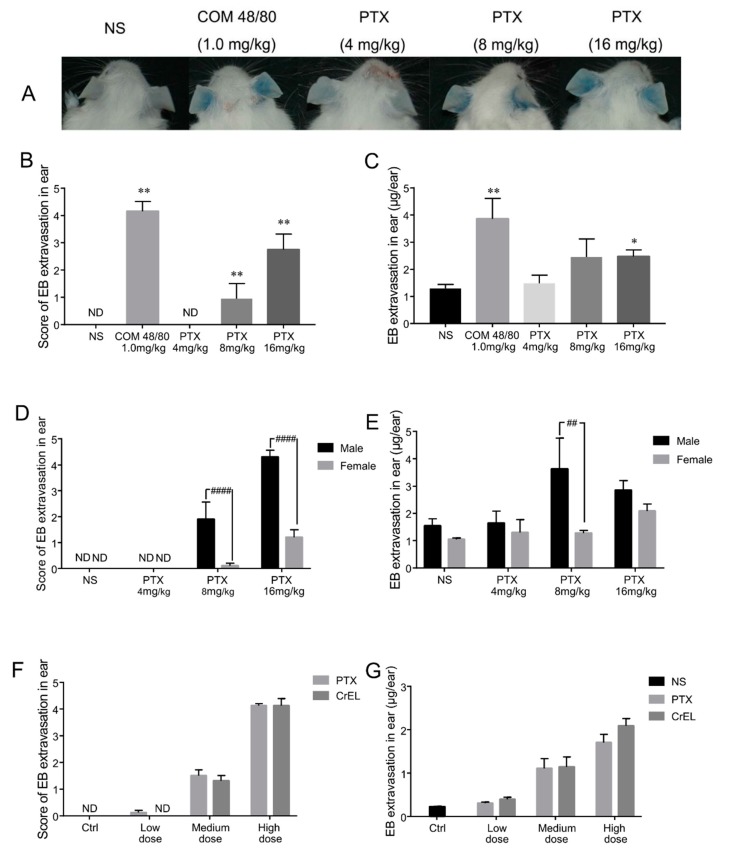
Paclitaxel injection induces vascular leakage in mice ears. (**A**) Blue color was observed in mice ears which received different treatments of normal saline (NS), compound 48/80 (COM 48/80) (1.0 mg/kg), or paclitaxel (PTX) injection (4, 8, or 16 mg/kg) plus Evans blue (EB). (**B**,**C**) The scores of blue colors and EB extravasation in mice ears with the administration of NS, COM 48/80, and 4, 8, or 16 mg/kg PTX injection (five male and five female mice per group). (**D**,**E**) Gender difference in scores and EB extravasation. (**F**,**G**) Vascular leakage of PTX injection and the excipient (CrEL) treatment (*n* = 8 male mice per group). Low dose, 4 mg/kg PTX injection or 3.33% CrEL, medium dose, 8 mg/kg PTX injection or 6.67% CrEL, and high dose, 16 mg/kg PTX injection or 13.33% CrEL, respectively. ND, the main score was 0 in the group. * *p* < 0.05 and ** *p* < 0.01 compared with the NS-treated group. ## *p* < 0.01 and #### *p* < 0.0001, comparison between male and female mice.

No visual blue-staining was observed in mice treated with normal saline/EB. Positive substance compound 48/80 (COM 48/80), a mast cell degranulation inducer caused strong ear-bluing reactions. The result showed that the blue color in ears was observed in a dose-dependent manner about 10 min after paclitaxel dosing (Figure 2A). The mean score of blue areas in the ear was 0, 0.95, and 2.75 in the 4, 8, and 16 mg/kg paclitaxel injection groups, respectively (Figure 2B). Vascular leakage in the ear was also assessed by the quantity of EB exudation at 30 min after the administration of drugs. EB exudations in 4, 8, and 16 mg/kg paclitaxel groups were 14.2%, 89.9%, and 91.4% higher than the negative control group, respectively (Figure 2C). Paclitaxel injection-induced ear vascular leakage in male mice was stronger than that in females (Figure 2D,E). Thus, male mice were employed in further experiments. Male mice were more sensitive than females. This may be associated with mast cell secretion regulated by progesterone [20], however, gender difference in human has yet to be clarified.

Next, paclitaxel injection and the excipients in paclitaxel injection (polyoxyl 35 castor oil and anhydrous ethanol, 1:1 *v*/*v*) were tested. When the concentration of paclitaxel in the injection was 4, 8, and 16 kg/mg, the relevant volume fractions of excipients were 3.33%, 6.67%, and 13.33%, respectively. The vascular leakage induced by merely excipients is equivalent to that evoked by paclitaxel injection under the same conditions (Figure 2F,G). It is suggested that in this study, the HSRs of paclitaxel injection was mainly caused by the excipients.

The above results indicate that the first exposure of paclitaxel injection could induce HSRs with manifestations on skin. These vascular leakage reactions were in a dose-dependence manner and mainly caused by relevant excipients in paclitaxel injection. Although polyoxyl 35 castor oil plays a crucial role in the occurrence of HSRs, this type of paclitaxel injection is still the most commonly used in China. Our research focused on the adverse effects caused by paclitaxel injection and hopes to provide a possible way to prevent HSRs in clinical practice, so the following experiments were carried out using paclitaxel injection rather than polyoxyl 35 castor oil.

### 2.2. Histamine Release Contributes to Paclitaxel-Induced Hypersensitivity Reactions

**Figure 3:**
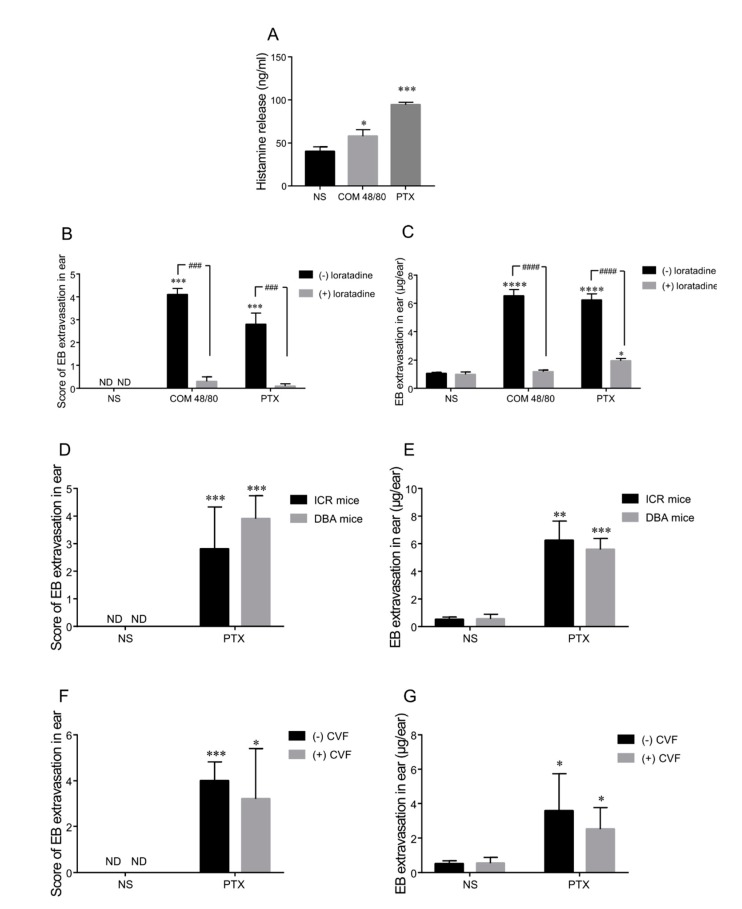
Effects of histamine release and complement activation on PTX injection-induced hypersensitivity reactions. (**A**) Histamine release in Institute of Cancer Research (ICR) mice serum (*n* = 5 male mice per group). (**B**,**C**) Vascular permeability in ICR mice after PTX injection administration with or without pretreatment with loratadine (*n* = 10). (**D**,**E**) PTX injection-induced vascular hyperpermeability in complement factor 5 (C5)-deficient mice (DBA) and ICR mice (*n* = 10). (**F**,**G**) With or without complement depletion pretreated by a Cobra venom factor (*n* = 10). ND indicted ear bluing score = 0. * *p* < 0.05, ** *p* < 0.01, *** *p* < 0.001, and **** *p* < 0.0001, comparisons were made in the treatment group vs. the NS group; ### *p* < 0.001, and #### *p* < 0.0001, with vs. without loratadine.

To verify the effect of paclitaxel injection on histamine release, plasma was collected 5 min after the intravenous injection of normal saline, COM 48/80, or a high concentration of paclitaxel injection in ICR mice. COM 48/80 is a G protein activator which induces mast cell degranulation and promotes histamine release. The histamine level in the positive control group (COM 48/80) increased significantly (*p* < 0.05) and was 45% higher than that in the negative group (normal saline). Paclitaxel injection also caused rapid histamine release and the increased concentration of blood histamine reached 2.4-fold compared with the normal saline group (*p* < 0.001, Figure 3A). These data suggest that histamine release was an early event involved in paclitaxel injection-induced HSRs.

Next, we evaluated the vascular leakage by treatment with 3 mg/kg of the anti-histamine drug, loratadine (an H1 receptor antagonist) prior to the administration of paclitaxel injected into ICR mice. The reaction rate of ear vascular leakage decreased from 100% to 20% by pretreatment with loratadine in the COM 48/80 and paclitaxel groups. A significant reduction of the average score of blue area in the ears could be observed as well (Figure 3B). In addition, EB exudation was dramatically inhibited by anti-histamine treatment; 82% and 69% lower than that without loratadine in the COM 48/80 and paclitaxel group, respectively (Figure 3C).

In summary, histamine released rapidly in mice plasma after COM 48/80 and paclitaxel injection treatment. On the other hand, the vascular leakage was markedly inhibited by pretreatment with anti-histamine reagent, confirming that histamine release was involved in paclitaxel injection-induced HSRs in mice.

### 2.3. Complement Is Not the Main Mediator of Paclitaxel Injection-Induced Hypersensitivity Reactions in Mice

We used two complement-deficient mice models, complement factor 5 (C5)-deficient mice (DBA/2N) and Cobra venom factor (CVF) complement-depleted mice, to further investigate the role of the complement pathway in paclitaxel injection-induced HSRs.

No obvious difference in vascular hyperpermeability was observed between DBA/2N and ICR mice, as assessed by the ear blue score and EB exudation (Figure 3D,E). Furthermore, CVF, a complement-activating protein which was shown to be a structural and functional analog of complement C3, was intraperitoneally injected into ICR mice to deplete total complement. Total complement could not be detected in CVF-treated mice and be detectable in control (non-CVF-treated) mice, which shows that CVF abolished total complement activity. However, vascular reactions induced by paclitaxel injection were nearly equal in CVF- and non-CVF-treated mice (Figure 3F,G). These results indicate that complement might not be a major factor of paclitaxel injection-induced HSRs in mice.

### 2.4. Paclitaxel Injection-Induced Hypersensitivity Reactions Were Associated with RhoA/ROCK Signaling Pathway Activation

Vascular leakage and edema induced by paclitaxel injection are related to vascular permeability. It has been reported that the RhoA/ROCK signaling pathway is associated with endothelial hyperpermeability [21]. The upregulated small GTPase RhoA leads to increased activity of Rho-associated kinase (ROCK) [22]. The activation of ROCK could increase the phosphorylation of the myosin light chain (MLC) and suppress the activity of myosin light chain phosphatase (MLCP) by phosphorylating the myosin binding subunit (MYPT). This could subsequently induce actin cytoskeleton reorganization and vascular endothelial hyperpermeability [23]. Therefore, we next verified the possible contribution of this pathway in paclitaxel injection-induced vascular leakage. 

**Figure 4:**
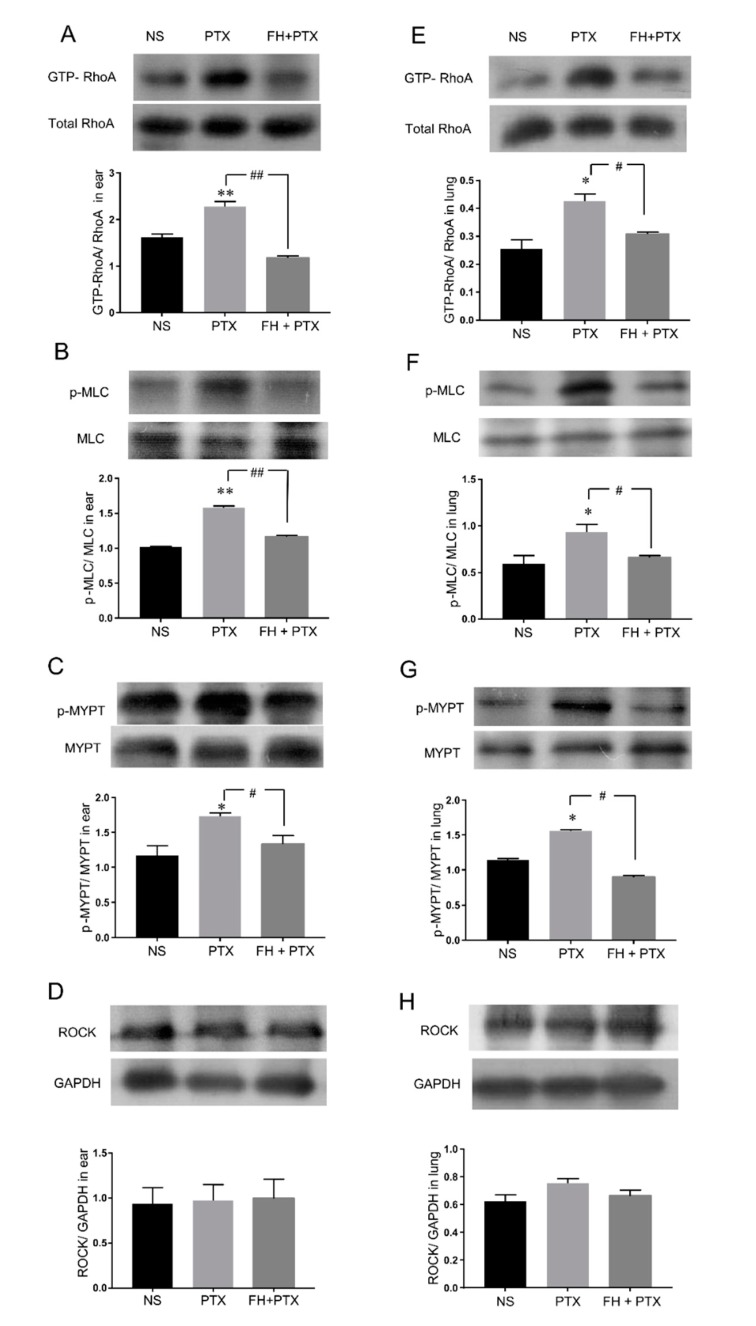
The inhibitory effect of fasudil hydrochloride (FH) on upregulation of key proteins in RhoA/ROCK pathway induced by PTX injection in mice ear (**A**–**D**) and lung (**E**–**H**) (*n* = 3 per group). (**A**,**E**) GTP-RhoA/total RhoA in mice ear or lung with PTX injection (16 mg/kg, iv) in the presence or absence of FH (30 mg/kg, ip). (**B**,**F**) p-MLC/MLC expression. (**C**,**G**) p-MYTP/MYPT value. (**D**, **H**) ROCK/GAPDH. * *p* < 0.05, and ** *p* < 0.01, compared with the NS-treated group, # *p* < 0.05 and ## *p* < 0.01 represented a significant difference between with and without FH pretreatment.

The results show that 16 mg/kg paclitaxel injection could obviously activate the RhoA/ROCK signaling pathway. The expressions of GTP-RhoA, phospho-myosin light chain (p-MLC), and phospho-myosin phosphatase targeting subunit 1 (p-MYPT) were significantly increased in the ears and lungs of mice (Figure 4A–H).

We next analyzed whether the specific inhibition of the RhoA/ROCK signaling pathway would attenuate paclitaxel injection-induced RhoA/ROCK activation. The data indicate that fasudil hydrochloride, a broadly used inhibitor of ROCK [24], inhibited augmentation of key proteins in the RhoA/ROCK pathway mentioned above. Pretreatment of intraperitoneal injected (ip) 30 mg/kg fasudil significantly reduced paclitaxel injection-induced upregulation of GTP-RhoA, p-MLC, and p-MYPT in the ears and lungs of mice (Figure 4A–H).

These results suggest that the activation of the RhoA/ROCK signaling pathway was involved in paclitaxel injection-induced HSRs.

### 2.5. Fasudil Inhibits Paclitaxel Injection Hypersensitivity Reactions in Mice

**Figure 5:**
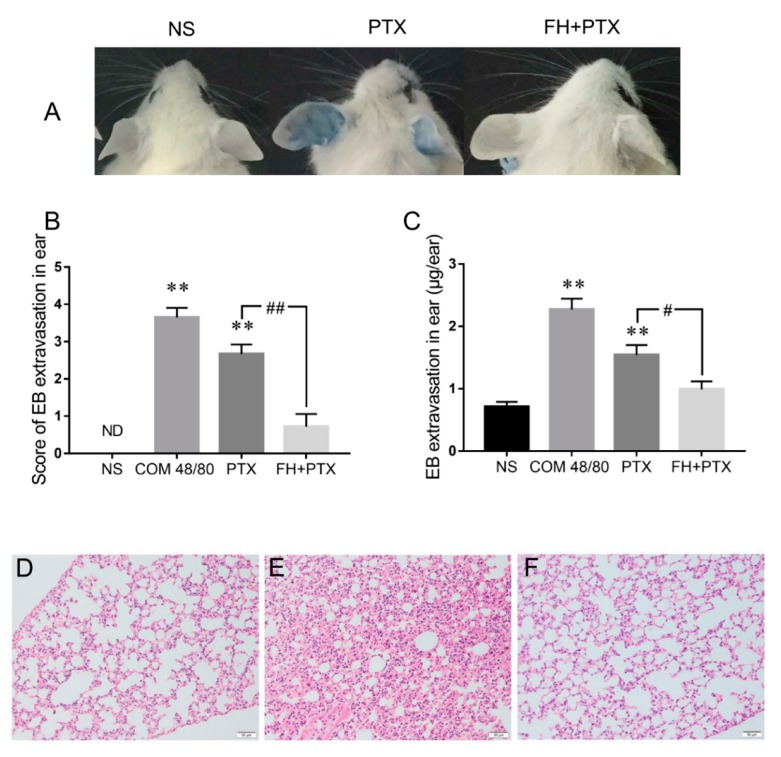
The inhibitory effect of FH on PTX injection-induced vascular leakage in mice ear and on lung inflammation. (**A**) The blue color in mice ears that received treatment of NS, PTX (16 mg/kg), and FH (30 mg/kg) plus PTX (16 mg/kg) injection. (**B**) Score of EB extravasation in the ear (*n* = 10). (**C**) EB extravasation in the ear (*n* = 10). ** *p* < 0.01, compared with the NS-treated group. # *p* < 0.05 and ## *p* < 0.01 represented the significant difference between the PTX injection single treatment and the concomitant FH pretreatment. (**D**–**F**) Microscopic examination of lungs, 200×. (**D**) NS, (**E**) PTX (16 mg/kg), and (**F**) FH (30 mg/kg) plus PTX (16 mg/kg) injection.

We further confirmed the effect of fasudil hydrochloride on paclitaxel injection-induced HSRs. The results showed that pretreatment with fasudil significantly attenuated vascular leakage in mice ears, by which the ear-bluing score was downregulated by 73.0% and the EB exudation was significantly decreased compared with the paclitaxel injection group (Figure 5A–C). Histological observations were performed to evaluate whether pretreatment with fasudil mitigated edema and inflammatory exudation in mice lung caused by paclitaxel injection. Histological changes, including congestion, edema with alveolar septal broadening, perivascular, and peribronchial monocyte infiltration were observed in the lungs of the 16mg/kg paclitaxel group. Consistently with ear vascular leakage results, pathological abnormalities were largely eliminated in the fasudil pretreatment group (Figure 5D–F). These results indicate that pretreatment with fasudil could notably inhibit paclitaxel injection-induced HSRs in the mice model.

## 3. Discussion

A high incidence of severe HSRs induced by paclitaxel infusion leads to the discontinuation of cancer chemotherapy [25]. These reactions occur immediately at the first exposure without prior sensitization [4]. Previous studies reported that the major effects of HSRs during paclitaxel infusion were attributed to histamine release and complement activation [10], yet this explanation is not sufficient to account for premedication failure with a combination of H1, H2 antagonist anti-histamines, and high-dose corticosteroids [13]. To date, the mechanism of paclitaxel injection-induced HSRs is still not completely understood. Developing more effective approaches to prevent HSRs remains a big challenge. 

The skin and respiratory manifestations, such as angioedema, generalized urticaria, and dyspnea, are the common symptoms of paclitaxel injection-induced HSRs and are correlated with vascular hyperpermeability. In the present study, we developed a mice model which could mimic the symptoms of vascular leakage to support the in vivo data of paclitaxel injection-induced HSRs and investigate the underlying mechanisms. We confirmed that this mice model clearly reacted in response to paclitaxel injection stimuli in a dose-dependent manner in the first exposure. In addition, this animal model was suitable to study allergic reactions induced by penicillin [26] and Shuanghuanglian injection [27] and has the possibility to become a practical method predicting other injections’ HSRs during the preclinical studies.

In this study, a rapid release of histamine was detected in mice serum after paclitaxel first exposure. The anti-histamine drug loratadine ameliorated paclitaxel injection-induced HSRs, indicating that these reactions were associated with histamine release. 

Due to poor solubility, paclitaxel was formulated in a special vehicle, polyoxyl 35 castor oil and anhydrous ethanol. We indicated that HSRs were mainly induced by the excipients in paclitaxel injection, which was in accordance with other researchers [9]. Complement activation can generate the anaphylatoxins C3a and C5a, which can interact with the G protein-coupled receptors C3aR and C5aR to mediate HSRs [28]. Based on the mechanism described above, paclitaxel injection would scarcely or rarely produce such anaphylatoxins under conditions of complement deficiency. However, in our results, C5-deficiency or total complement depletion could not alleviate HSRs. Vascular hyperpermeability in both of the complement-deficiency mouse models was comparable with that that occurred in normal mice. Our study revealed that complement activation might not be the primary mechanism of paclitaxel injection-induced HSRs. These findings conflict with the results of in vitro data [29] and further studies are required for validation in the future. 

The RhoA/ROCK signaling pathway has been implicated in immunoinflammatory diseases, such as systemic lupus erythematosus [30], myasthenia gravis [31], inflammatory cardiovascular diseases [32], and inflammatory airways diseases [33]. In addition, the RhoA/ROCK pathway also involves endothelial hyperpermeability [26] and mediates histamine-induced vascular leakage [14]. Therefore, we hypothesized that the RhoA/ROCK signaling pathway might be associated with paclitaxel injection-induced HSRs. Our results demonstrate that paclitaxel infusion upregulated the expression of GTP-RhoA (active state RhoA) and p-MYPT (a hallmark of ROCK activity), resulting in an increase of p-MLC expression. The phosphorylation of MLC induces actin cytoskeleton reorganization and contraction, which leads to widening of endothelial intercellular space and increased vascular permeability [21,34]. As a result, plasma and blood cells effuse outside the blood vessels, causing tissue edema and exudative inflammation. Some research reported that paclitaxel could enhance the expression and activity of Rho-kinase in human coronary artery smooth muscle cells [35], which is consistent with our results. ROCK inhibitor fasudil has a significantly protective effect on paclitaxel injection-induced vascular leakage and pathological change in the ears and lungs of mice by decreasing GTP-RhoA, p-MYPT, and p-MLC expression. Our study indicated that activation of the RhoA/ROCK signaling pathway played an important role in paclitaxel injection induced-HSRs. 

The inhibitory role of nitric oxide (NO) via cGMP in the RhoA/ROCK pathway has been reported [36,37]. On the other hand, some NO donors such as the antiretroviral protease inhibitors lopinavir-NO [38,39] and ritonavir-NO [40] are emerging as the potential candidates for an anticancer drug with a more potent chemotherapeutic effect. These present findings may open new investigations on the concomitant medication of NO-donor chemotherapeutic agents and paclitaxel to prevent RhoA/ROCK-dependent HSRs while possibly synergizing chemotherapeutic efficacy.

In conclusion, a suitable and practical in vivo mice model, characterized by vascular hyperpermeability and exudative inflammation and mimicking HSRs was developed in our study. Polyoxyl 35 castor oil, the main excipient in paclitaxel injection, was associated with severe vascular leakage. Histamine release and RhoA/ROCK pathway activation, rather than complement activation, play an important role in paclitaxel injection-induced HSRs. Furthermore, the ROCK inhibitor may provide a potential preventive approach for paclitaxel injection side effects.

## 4. Materials and Methods 

### 4.1. Animals

All the animal studies were carried out in accordance with the recommendations of the ethical guidelines and regulations for the use of laboratory animals issued by the Laboratory Animal Welfare Ethics Committee in the Institute of Chinese Materia Medica, China Academy of Chinese Medical Sciences. All animal-related procedures (the project identification code is 20162004, date is 15 February 2016) adhered to the protocol were approved by the same committee mentioned above.

ICR mice (male and female) and DBA/2N mice (male) were purchased from Vital River Laboratories Animal Technology (Beijing, CHN) at 8–10 weeks of age and randomly divided into experimental groups according to body weights. Animals were kept under specific pathogen-free conditions.

### 4.2. Reagents and Antibodies

The following reagents were obtained from the indicated suppliers: Paclitaxel injection (5 mL: 30 mg, the excipients in the paclitaxel injection are polyoxyl 35 castor oil, anhydrous citric acid and anhydrous ethanol, Beijing Union Pharmaceutical Factory, Beijing, CHN), relevant vehicle (polyoxyl 35 castor oil and anhydrous ethanol, 1:1 *v*/*v*, Beijing Union Pharmaceutical Factory, Beijing, CHN), compound 48/80 (Sigma-Aldrich, Louis, MO, USA), Evans blue (Sinopharm Chemical Reagent Co., Ltd., Shanghai, CHN), and Cobra venom factor (Shanxi Powerdone Pharmaceutics Co., Shanxi, CHN).

The following antibodies were used in this study: anti-p-MYPT1 (Thr 696) (rabbit polyclonal, 5163, Cell Signaling Technology (CST), MA, USA); anti-MYPT1 (rabbit polyclonal, 2634, CST, MA, USA), anti-p-MLC2 (Thr18/Ser19) (rabbit polyclonal, 3674, CST, MA, USA), anti-MLC2 (rabbit polyclonal, 3672, CST, MA, USA), Anti-RhoA (67B9) (rabbit monoclonal, 2117, CST, MA, USA), anti-ROCK1 (rabbit monoclonal, EP786Y, Abcam, Cambridge, UK), anti-GAPDH (rabbit polyclonal, FL-335, Santa Cruz Biotechnology, CA, USA) and the secondary goat anti-rabbit IgG (H + L) antibody (ZSGB-BIO, Beijing, CHN).

### 4.3. Assessment of Vascular Leakage in ICR Mice

ICR mice were randomly divided into different groups on a body weight-stratified basis. Mice were treated by slow intravenous injection of normal saline, COM 48/80 (1.0 mg/kg), paclitaxel injection (4, 8, and 16 mg/kg) or relevant vehicle (polyoxyl 35 castor oil and anhydrous ethanol, 1:1 *v*/*v*) (3.33%, 6.67%, and 13.33%) through the tail vein (0.1 mL/10g), and successively injected (iv) an equal volume of 0.8% EB dissolved in normal saline. The concentration of the relevant vehicle corresponded to the low, medium, and high dose of paclitaxel injection. EB performance was to visualize the ear blue color and quantify the extent of vascular leakage in ears, so as to assess the skin manifestation of paclitaxel injection-induced HSRs.

Thirty minutes after drug/EB treatment, vascular leakage was assessed by observation of the blue color in mice ears and evaluated by giving a score of 0 to 5 according to the blue color areas in the ear, where “0” represented no visible blue area, and “1 to 5” denoted visible blue area ratio of < 1/8, 1/8 to 1/4, 1/4 to 1/2, 1/2–3/4, and >3/4, respectively. In order to ensure the reliability and reduce the subjective difference, the same researcher was appointed to score the blue color area, blinded to the treatment of mice as well. Subsequently, the ears of each mouse were shredded and preserved in 2 mL of formamide for EB extraction. The lungs of each mouse were maintained in formalin for histopathological examination.

### 4.4. Histamine Assays and Anti-Histamine Treatment in ICR Mice 

ICR mice were randomly divided into normal saline, COM 48/80, and paclitaxel groups and each group had five male ICR mice. One heparin-anticoagulative blood sample from each mouse was collected 5 min post-injection. Plasma was prepared by centrifugation for 15 min at 3500 rpm. Histamine concentrations were assayed by enzyme-linked immunosorbent assays (ELISA, Immuno-Biological Laboratories Inc., Minneapolis, MN, USA) according to the manufacturer’s instruction.

Parallel groups were used for anti-histamine experiments. Five male ICR mice in each group received normal saline, COM 48/80, or a paclitaxel injection combined with EB as before, while another five male ICR mice received oral 3 mg/kg loratadine twice at 0.5 and 5.5 h prior to administration [41]. Ear vascular permeability was assessed as described in the Section entitled “Assessment of Vascular Leakage in ICR mice”.

### 4.5. Vascular Permeability Assay in Complement-Depleted Mice

Two models were used for complement depletion. 1) Complement-depletion model: CVF was given to ICR mice and total serum hemolytic complement activity was assayed to confirm complement depletion [42]. ICR mice were randomly divided into normal saline and paclitaxel groups. Half of the mice in each group received two times of CVF (ip, 0.1 mL/10g, 37.5 U/mL CVF in saline). The first injection was administered 24 h before drug administration, and the second injection was given 5 h after the first CVF injection [43]. The mice in the other half of each group without CVF treatment were intraperitoneally administered the same volume of normal saline. The time frame for total serum hemolytic complement activity detection was coincident with paclitaxel infusion in relation to CVF treatment. 2) Complement-deficiency model: In DBA/2N mice, parallel groups that were not pretreated with CVF were used. These were deficient for C5. EB was intravenously injected into mice to assess ear vascular permeability as described above. 

### 4.6. Paclitaxel Injection Induced RhoA/ROCK Signaling Pathway Activation

Mice were treated (iv) with normal saline, 16 mg/kg paclitaxel injection or pretreated with 30 mg/kg fasudil (ip) and then received (iv) 16 mg/kg paclitaxel injection. Ears and lungs were removed 30 min after dosing and were homogenized with RIPA lysis buffer. Supernatants of lysates were collected after centrifugation and stored at −80 °C for western blot assays. Samples were separated by SDS polyacrylamide gel electrophoresis. Proteins in the gels were transferred onto PVDF membranes and then blocked with skimmed milk at room temperature for 2 h. Membranes were incubated with primary antibodies of anti-p-MYPT1, anti-MYPT1, anti-p-MLC2, anti-MLC2, anti- RhoA, anti-ROCK1, or anti-GAPDH at 4 °C overnight. After being washed by TBST, membranes were incubated in secondary goat anti-rabbit IgG antibody at room temperature for 2 h and visualized by enhanced chemiluminescence detection substrate. GTP-RhoA was assessed by a pull-down assay (Cytoskeleton, Denver, CO, USA) according to the manufacturer’s instructions. We used ImageJ software to analyze blots Images.

### 4.7. Effect of ROCK Inhibitor on Paclitaxel Injection-Induced Hypersensitive Reactions

Mice were intraperitoneal injected with 30 mg/kg fasudil once daily for three consecutive days. At 30 min after the last dosing of fasudil, mice received (iv) 16 mg/kg paclitaxel injection/EB. In the parallel group, mice were treated (iv) with only 16 mg/kg injection and EB solution consecutively. After thirty minutes, the ear score was evaluated. The ears of each mice were immersed in 2mL of formamide for EB extraction and the lungs were preserved for histopathology analysis.

### 4.8. Statistical Analyses

Data were expressed as mean (M) ± standard error of the mean (SEM). Quantitative data were analyzed using a one-way analysis of variance method. The score of vascular leakage of the ear was analyzed with a rank test. Statistical analysis was performed with SPSS 16.0 software. A *p*-value of less than 0.05 was considered significant.

## Abbreviations

HSRs	Hypersensitivity reactions
IgE	Immunoglobulin E
iv	Intravenous injection
ip	Intraperitoneal injection
EB	Evans blue
NS	Normal saline
COM 48/80	Compound 48/80
PTX	Paclitaxel
CVF	Cobra venom factor
C3	Complement component 3
C5	Complement component 5
ELISA	Enzyme-linked immunosorbent assays
